# Peer Review of “Exploring the Reasons for Low Cataract Surgery Uptake Among Patients Detected in a Community Outreach Program in Cameroon: Focused Ethnographic Mixed Methods Study”

**DOI:** 10.2196/38873

**Published:** 2022-06-09

**Authors:** Jo-Hsuan Wu

**Affiliations:** 1 Department of Ophthalmology University of California San Diego La Jolla, CA United States

**Keywords:** ophthalmologic surgical procedures, access to health care, ophthalmology, patient-centered care, ethnography, health knowledge, attitudes, practice

*This is a peer-review report submitted for the paper “Exploring the Reasons for Low Cataract Surgery Uptake Among Patients Detected in a Community Outreach Program in Cameroon: Focused Ethnographic Mixed Methods Study.”*

## Round 1 Review

### General Comments

This is an interesting qualitative study assessing the current status and potential causes underlying the low uptake of cataract surgery among community-diagnosed patients with cataract in Cameroon [1]. The authors showed extensive knowledge about the context of the study and provided valuable suggestions on how health care professionals may help solve the problem. The interview was detailed, and the methodology was well designed and clearly explained.

While the topic has its merits and the discussion is quite comprehensive, I believe the manuscript can be further improved after some points are addressed or some questions are clarified.

### Major Comments

1. The lengths of both the main text and the abstract are a bit long. We suggest the authors to further condense the paper or move some parts to Multimedia Appendices.

2. Although 29 subjects were interviewed, only 9 of them were direct subjects. We are unsure if this is a sufficient number for such qualitative analysis.

3. The influence of indirect subjects' opinions on the decision of the direct subjects was not particularly discussed.

4. Considering the potentially different weights of direct versus indirect subjects' opinions in the decision, whether the quotes were taken from direct subjects should be shown.

5. We are no experts of traditional medicine, but is there anything to be noted about these therapies? (Maybe certain therapies were helpful from the patients' perspectives?) We are unsure if these should be taken into consideration when assessing the “Knowledge and awareness” and “reasons of refusal.”

6. The “poor outcome” of prior cataract surgeries was mentioned in the *Results* section. Can this be a possible reason for the “fear” of cataract surgery and the reason to choose traditional medicine instead?

### Minor Comments

7. There are still some grammatical mistakes that should be checked and amended.

8. Please make sure to provide the full spellings of all abbreviated words at first use (eg, “MICEI” and “FGDs”).

9. The table did not show the particular demographics of the direct subjects (which may help reveal other socioeconomic factors influencing the decision or limitation of the study).

10. How is the surgery acceptance or backlog situation for community cataract screening programs conducted in nearby countries with a similar socioeconomic status? While this is not the focus of the study, if there are available data, it would be good to include some general information (this will help justify the study aim and support the overall results).

## Round 2 Review

### General Comments

The authors have addressed most of the comments. While the scientific content is acceptable after the revision, it is still recommended that the authors shortened the article to <6500-7000 words. No further suggestions are enclosed.
